# “We are Not Magicians”: Midwives’ Experience of Stillbirth in Nigeria

**DOI:** 10.1177/00302228251327719

**Published:** 2025-03-13

**Authors:** Tosin Popoola

**Affiliations:** 1 School of Nursing and Midwifery, 5982 The University of Newcastle, Callaghan, Australia

**Keywords:** Africa, midwives, Nigeria, stillbirth, stillbirth bereavement

## Abstract

Experiencing stillbirth in midwifery practice has a profound impact on midwives. However, midwives’ experience of stillbirth remains under-explored, especially in Nigeria, where the burden of stillbirth remains one of the highest. This study describes the experiences of Nigerian midwives who have experienced a stillbirth in their midwifery practice. Data were collected using semi-structured interviews with 11 midwives between December 2022 and January 2023. Interviews were transcribed verbatim to explore midwives’ experience of stillbirth and analysed using the template analysis technique. Three key themes were identified: (1) emotional and psychological reactions to stillbirth, (2) the impact of stillbirth on midwives, and (3) the support needs of midwives after a stillbirth. This study highlights that stillbirths strike at the core of professional identity and lead to negative self-concept and cautious-based practice. However, the working conditions of midwives in Nigeria do not support them to deal with the multidimensional impact of stillbirth.

## Introduction

During the period between pregnancy, childbirth, and postnatal, pregnant women and midwives work in partnership to achieve the best possible outcomes for the mother and child (International Confederation of Midwives [ICM], 2014). This period is often exciting as relationships develop between parents expecting their newborns and midwives working to ensure the pregnancy experience is positive for parents. However, while midwifery practice is focused on facilitating the arrival of new life, not all pregnancies end in a live birth. Almost two million babies are stillborn every year (UNICEF, 2020). Stillbirths shatter parents’ dreams and change the focus of midwifery care from essential newborn care to bereavement care (Yenal, Tektaş, Dönmez, & Okumuş, 2023). The replacement of essential newborn care with bereavement care can be overwhelming and confronting for midwives who have been trained to care for a new life.

Stillbirths are severe adverse events because they result in the death of a baby. Evidence from previous research highlights that adverse events in healthcare primarily affect the patient, followed by the healthcare professionals involved in the incident (McDaniel & Morris, 2020). However, since parents are considered the entities that suffer the most after a stillbirth event, most research has focused on different aspects of stillbirth loss from the parental perspective. For example, research has focused on their experience (Bradford et al., 2024; Popoola et al., 2024), coping strategies (Alvarenga et al., 2021), the impact of stillbirth (Cena et al., 2021), and the need for quality bereavement care (Atkins et al., 2022; Popoola et al., 2022). Evidence from these studies reveals that stillbirth increases the risk of negative well-being, sleeping disturbances, income loss, marital disharmony, anxiety, and depression for parents (Cena et al., 2021; Sarkar et al., 2022).

The impact of stillbirth is, however, not limited to parents. Midwives’ position as the primary caregivers during pregnancy, childbirth, and the postpartum period exposes them to the raw emotions of parental grief when labour ends in the death of a baby. This firsthand experience of stillbirth comes with significant costs for midwives. For example, research reveals that the feelings of shame, guilt, uncertainty about professional abilities, anxiety and symptoms of post-traumatic stress disorder (PTSD) permeate the experience of midwives who have encountered stillbirth in their practice (Bingham et al., 2023; McDaniel & Morris, 2020; Mok et al., 2020; Ravaldi et al., 2022).

Despite the impact of stillbirths on midwives, research tends to focus on their bereavement care skills (Ravaldi et al., 2022; Sheehy & Baird, 2022), not on midwives’ own encounters with stillbirth. This dearth of research on midwives’ experience of stillbirth in midwifery practice is even worse in countries bearing the brunt of stillbirths. For example, in a country like Nigeria, where the rate of stillbirths is one of the highest in the world (UNICEF, 2020), no research has explored midwives’ stillbirth experiences. This is significant because midwives in low and middle-income countries (LMICs) like Nigeria often have limited resources to deal with the impact of stillbirth (Nachinab et al., 2021). This suggests that the impact of stillbirth may be higher on midwives in LMICs and may impact their ability to support parents faced with stillbirth.

### Stillbirth in Nigeria

Like many other social and public health issues, inequities exist in how stillbirth impacts each region. For example, Sub-Saharan Africa (SSA) region accounts for over half of all stillbirths in the world (UNICEF, 2020), and some countries in SSA, such as Nigeria, have stillbirth rates that are up to 23 times higher than those of high-income countries (UNICEF, 2020). In 2019, Nigeria recorded 171,428 stillbirths, making it the country with the third largest burden of stillbirth (UNICEF, 2020). The determinants of stillbirth in Nigeria are not dissimilar to those of high-income countries in that women of lower socioeconomic status bear a disproportionate burden. As an illustration, living in squalid conditions, living in remote areas, limited or no formal education, lack of antenatal care, prolonged labour, maternal hypertensive disorders, and birth complications are the main determinants of stillbirth in Nigeria (Okunowo & Smith-Okonu, 2020).

However, the true burden of stillbirths in Nigeria is hard to quantify because of the pluralistic maternal healthcare system, which is made up of a significant amount of unregulated health providers. As an illustration, unskilled and unregulated providers assist over 50% of births in Nigeria outside of a health facility (National Population Commission, 2014). Parents’ reliance on unregulated and informal health providers for maternity services despite the associated risks has been blamed on the government’s under-investment in the orthodox healthcare system in Nigeria (Agwu et al., 2024). However, pregnancy outcomes in the hands of informal and unregulated health providers are often missed in the official health statistics, leading to under-reporting of pregnancy outcomes, including stillbirths.

Data from the 2013 Demographic and Health Survey in Nigeria shows that 63% of women give birth at home, and 45% are assisted by relatives or traditional birth attendants (National Population Commission, 2014). When complications arise during these homebirths, it is often too late to save the babies by the time the woman makes it to a health facility. The consequence of this is that half of the stillborn babies in Nigeria die after labour has started (UNICEF, 2020). Arrival in a health facility, even when it is too late to intervene to save the lives of the babies, can still reflect poorly on midwives, as it is not uncommon for parents to think that merely reaching the health facility should be enough to save the baby. This is not surprising. According to McKellar et al. (2019), the public thinks of midwives when they hear ‘birth’. In the event of a stillbirth, parents may perceive that a midwife has not lived up to their public persona, which might lead to many challenges, such as blame, accusations of negligence, and legal issues (Austin et al., 2014).

Understanding midwives’ experiences of stillbirth from the perspective of Nigerian midwives is essential not just because of the country’s high burden of stillbirth but also because midwives in low-income countries tend not to have the institutional support required to deal with stillbirths in their practice. For example, studies conducted in Ghana (Nachinab et al., 2021) and Lao (Choummanivong et al., 2020) found that midwives had no organisational support to manage their personal feelings after experiencing a stillbirth in practice. This suggests that the impact of stillbirth on midwives in low-income contexts might be more significant. Therefore, this study aimed to describe the experiences of Nigerian midwives who have experienced a stillbirth in their midwifery practice. The research question was: How do Nigerian midwives experience the death of a baby due to stillbirth?

## Method

A qualitative descriptive design was employed to describe the participants’ experience of stillbirth. Qualitative description helps with understanding the ‘who' (midwives), ‘what' (experience of stillbirth in midwifery practice), and ‘where' (Nigeria) components of the research problem (Doyle et al., 2020), all of which are important to understand the findings.

### Study Setting and Recruitment

This study was conducted across multiple settings of regulated midwifery practice in Nigeria. Eleven registered midwives working in regulated facilities in Nigeria and who had experienced a stillbirth in their practice were interviewed by phone between December 2022 and January 2023. Participants were identified through the author’s professional networks. Study advertisements were sent to the author’s professional colleagues in Nigeria through email, and these colleagues then shared information about the study with their networks. Interested midwives contacted the author through email, and the author followed up with further information to assess their understanding of the study and determine their eligibility. Thereafter, the participants were sent the information sheet and informed consent forms before the phone interviews were conducted.

### Data Collection

Data were collected with open-ended, semi-structured interviews that ranged between 22 to 43 minutes. During the eligibility check, the author perceived that some participants wanted an idea of what they might be asked during the interview. As such, the sample interview guide was sent to the participants beforehand to lessen their anxiety. The interview questions were structured around four key topics: (a) a childbirth that ended in stillbirth; (b) emotions associated with conducting births that end in stillbirth; (c) the impact of conducting births that end in stillbirth, and (d) coping with births that end in stillbirth. The interview questions were developed based on earlier studies (Austin et al., 2014; Jones & Smythe, 2015; McDaniel & Morris, 2020) and were peer-reviewed during the ethics review process. All the interviews were audio-recorded with the approval of the participants. TP, a Nigerian native and PhD-prepared registered nurse and midwife, conducted the interviews in English.

### Data Analysis

Interview data were analysed using the systematic method of Template Analysis. Template Analysis (TA) is a form of qualitative analysis where a coding structure (template) is developed and applied to the data in the analysis process (Tabari et al., 2020). In TA, the analysis starts with identifying a priori codes, which are codes identified before the main analysis due to their relevance to the issue being studied. The procedure for using TA for qualitative analysis is flexible. However, it generally follows a six-step process (Tabari et al., 2020) that starts with data familiarisation and ends with writing up (Table 1). In this study, 112 a priori codes strongly relevant to the research question were first identified from the summary notes written at the end of every interview. These priori codes and the transcripts were then imported into NVivo software, where data familiarisation, coding and the development of the final template were completed. During the reading of each transcript, the priori codes (template) were modified by identifying new codes, refining existing codes, or eliminating irrelevant codes and applied to the whole dataset. The final template constituted the study findings, which are the themes and the sub-themes representing how midwives experience stillbirth in Nigeria.

**Table 1.** Stages of Template Analysis.

1	Data familiarisation	Each transcript was checked for accuracy
2	Preliminary coding of data	A priori codes were identified during the writing of interview summaries and reflections. For example, the impact of stillbirth, such as negative self-concept, was recurring after every interview
3	Organising the emerging themes into meaningful clusters	Increased understanding of the data enabled interpretations of the relationships between codes and thematic groupings. For example, the impact of stillbirth on midwives was organised around the personal, professional and organisational dimensions
4	Defining an initial coding template	The initial thematic structure was based on the first four interviews and selected based on the resonance across the data as a whole
5	Applying the initial data to further data and modifying as necessary	The initial template was applied to the remainder of the data, which led to the confirmation of the enduring themes and revising and reorganising the themes
6	Finalising the template and applying it to the data set	Repeated data coding continues to ensure that no data relevant to the research question remains uncoded

### Ethical Considerations

Ethics approval was received from the Human Ethics Committee of Victoria University of Wellington (Approval no. 30705). The participants participated in the study voluntarily by returning signed informed consent forms. Acronyms were used to anonymise the participants and present the findings.

### Rigour and Reflexivity

In qualitative research, rigour encompasses producing rich and credible findings (LaDonna et al., 2021). Thus, rigour requires deliberate decision-making in the research. In this study, rigour was achieved by seeking and obtaining feedback on the findings from the participants (confirmability), being reflexive on the limitations of the study (credibility), being transparent about the research procedures (dependability), providing sufficient information about the context (transferability), and supporting findings with sufficient quotes (credibility). TP, who conducted and analysed the data, also has experience in conducting research related to death and dying, including stillbirth (credibility).

## Results

Overall, 11 participants, all of whom were females (Table 2), participated in the study. Participants were between 36 and 55 years old, with the average age being 44.5 years. In line with the participants’ age, years of experience as a midwife ranged from 10 to 32 years, with the average years of experience being 19 years. All the participants have the basic qualifications required to practice midwifery in Nigeria: registered nurse (RN) and registered midwife (RM) certifications. The participants were from two of the biggest tribes in Nigeria: Yoruba and Igbo. The participants worked in different maternity settings, such as tertiary, secondary and primary-level care and private practice.

**Table 2.** Study Participants’ Characteristics (*n* = 11).

Participant	
Sex	
Female	11
Age in years	
Mean (range)	44.5 (36–55)
Working experience as midwives in years	
Mean (range)	19 (10–32)
Ethnicity	
Yoruba	8
Igbo	3
Settings of midwifery practice	
Tertiary hospital	4
General hospital	3
Private practice	2
Primary healthcare	1
Educational/professional qualifications	
RN, RM, BNSc	7
RN, RM	4
Current area of midwifery practice	
Labour ward	7
Postnatal ward	3
Primary health care	1
Number of stillbirths conducted since becoming a midwife	
Mean (range)	18 (1–85)

## Themes

Three main themes were identified: (1) emotional and psychological reactions to stillbirth, (2) the impact of stillbirth on midwives, and (3) the support needs of midwives after a stillbirth.

## Emotional and Psychological Reactions to Stillbirth

This theme describes three subthemes of emotional pain and sadness, guilt and shame and the factors compounding emotional and psychological reactions.

### Emotional Pain and Sadness

The participants experienced sadness and emotional pain whenever a stillbirth occurs in practice. Participants used strong emotional words to capture their experience of sadness:

… It is always sad when you have been with a woman from the first stage of pregnancy, taken care of her, formed a relationship with her and then at [birth], you birthed a flat [dead] baby…to me, it feels like a wasted time and effort for me as a midwife and for the woman because we have nothing to show for our efforts. (P10)

Birthing a live baby was integral to how participants saw themselves and as such, their grief was not just centred around the baby that died, but also on the partnership between them and the women that did not yield the intended outcome. As such, most participants said stillbirth turned the ‘joy of midwifery’ to sadness, as seen below:

At the end of every labour, no matter how challenging, we look forward to saying, ‘Congratulations, you have birthed a baby boy, baby girl or twins as the case may be’. That is the joy of every midwife…When you find yourself in a situation where you have to give the diagnosis of stillbirth to parents, the joy of midwifery is replaced with pain and sadness. (P5)

Losing the joy of midwifery highlights the emotional pain associated with stillbirth and the connection between personal and professional aspects of midwifery.

### Guilt and Shame

As the participants tried to make sense of the incident, some experienced guilt. Guilt featured strongly in their experience because midwives associated stillbirth with inadequate use of midwifery skills. Participants who felt responsible for the loss also experienced shame.

As a midwife, there are lots of emotions…to be specific, emotions of guilt, like you caused the stillbirth maybe due to negligence or something or maybe you did not do what you are supposed to do at the right time for the baby or the mother and the emotion of shame. (P6)

To a certain extent, feelings of guilt was associated with perceived culpability and whether the circumstances that led to the death were under the control of midwives.

I have never felt guilty because I always do my work. Whenever I [conduct a birth], I do everything expected of me as a midwife. I use my clinical judgement to decide the appropriate time to augment labour, and I check vital signs regularly. I don’t delay with my interventions…If the baby dies after following all the correct procedures, let’s say, from cord abnormalities, there is no guilt because there is nothing I could have done. (P2)

### Factors Compounding Emotional and Psychological Reactions

Witnessing parents’ display of raw emotions was challenging for midwives and, in many cases, modulated midwives' emotional and psychological state, as seen below:

…the father could not absorb the shock; he was weeping like a baby…I remember the grandmother telling me, “How can you say this plump baby is dead? Please help us and do something”. I was helpless and moved to the point of crying, but I still had to be there for them, so they don’t lose hope and fall into depression. (P5)

Unexpected stillbirths were sometimes accompanied by blame and dissatisfaction from parents.

Some parents show understanding and don’t blame us or cause a scene, especially when they see everyone running around to resuscitate the baby…But we also get a lot of blame and abuse. Some parents have said we should have taken the woman to the theatre for emergency C-Section. (P4)

Around August, we had a stillbirth that resulted in the assault of one of my colleagues. … the husband went into a rage and before we knew what was happening, one of the midwives got a dirty slap across her face (P8)

This shows that stillbirths can make the work environment unsafe and hostile for midwives.

## Impact of Stillbirth on Midwives

This theme describes three subthemes: (1) negative perceptions of self and profession, (2) changes in practice, and (3) enduring the inquisition.

### Negative Perception of Self and Profession

According to the ICM (2014), midwives are healthcare professionals trained to use their skills to conduct births, detect complications, and provide appropriate assistance necessary to ensure the safety of women and newborns. When midwives achieve good outcomes for women and their babies, they describe it as a proud moment in midwifery. However, when a baby dies under midwifery care, participants feel they have not measured up to their scope of practice, leading to a lack of satisfaction and fulfilment in performance.

…I would say it is not a proud moment in midwifery when a baby dies in your care. I had a woman admitted with a foetal heart rate of 130/140, and at the end of that [birth], the baby did not make it [died]…losing that child at the time of [birth] felt like a slap in the face; it felt as if we didn’t know what we were doing. (P4)

The feelings of humiliation that the participants experience after stillbirth are derived from the ethos of midwifery, which involves working in partnership with women, families, and other healthcare providers to ensure the delivery of quality care. In the event of stillbirth, the participants felt accountable to their patients, colleagues and the profession, which led to anxiety about the public’s perception of the event and its potential to put the profession in a negative light. One participant said, “*When it dawned on me that we had lost the baby, I remember feeling down and in my mind, I was thinking – this creates a bad image for midwives* (P10)”.

### Changes in Practice

The experience of stillbirth impacted the participants’ practice positively and negatively. Positively, it led to quality improvement initiatives, re-training and stillbirth prevention initiatives.

… We have had to re-train, and as a midwifery team, we have been at the forefront of advocating for more postmortems so that we can make necessary changes and learn from the event…Since most stillbirths also happen among women who delay before they come to the hospital, we have also started a community education and awareness programme to raise awareness about the dangers of delay. (P7)

Negatively, it led some participants to adopt a more cautious-based approach to midwifery practice, with many participants lamenting about its impact on positive birth experience.

I conducted one birth two months ago and gave a very generous episiotomy; suturing it back was a mess. I would typically not give that amount of episiotomy, but I didn’t want to take any chance since the scan showed the baby was big. In retrospect, I didn’t need to go to that extreme with my interventions, given that the mother was gravida 3 para 2 and had no complications in prior pregnancies … The baby was eventually 3 kg. (P9)

Such an approach could also involve a woman being referred on.

I have found myself referring more patients to the next level of care, and many end up with more interventions and caesarean sections. As a midwife, I feel bad when women don’t have normal births and when their eventual labour does not look like anything we have planned. (P8)

### Enduring the Inquisition

As stillbirths are not the normal nor desired outcomes of childbirth, all the participants had to answer queries about the stillbirth events. There were differences in how queries were administered to midwives depending on the settings they worked. For example, community and primary care midwives report stillbirth incidents to their superiors, and there was no escalation beyond their managers. In contrast, those working in private, secondary, or tertiary levels of care faced more stringent inquisitions, and many also faced a panel of experts who reviewed the care provided. Regardless of the process or practice setting, the experience was the same: anxiety-provoking and terrifying. However, the participants also said the inquisitions were important, with many saying they learned from the experience.

A query always accompanies stillbirths; you have to state everything that happened – the time of admission, how the patient came in, what happened, what you did when you suspected something was wrong and everything that happened throughout the labour. After you have answered the query, you will face a panel, and they will review what happened. There is always a lot of anxiety and fear, but it is also a moment of reflection and teaching. (P1)

The outcome from the panel can take time.

The first six months after the event, I was scared to death and living in fear. I didn't want to go back to work because I didn't know what the panel's recommendations would be – I didn't know whether that would be the end of my midwifery career. (P9)

The participants' narratives also revealed that the inquisition and the fear of consequences led to blame among healthcare providers, and parents were not spared.

Initially, the Medical Director, who is also a gynaecologist, blamed the midwives for poor observation and decision-making, especially with the use of Pitocin, but later on, the panel found that the woman had underlying gestational diabetes that her doctor did not pick up during pregnancy. The blame then shifted to the medical team...so it wasn’t the fault of the midwives but the doctors. (P1)

The woman was the cause of it because she [skipped] antenatal classes and was uncompliant with medical advice. (P2)

In addition to being terrified of the formal process of inquiry, there were also informal practices that put a spotlight on midwives who had lost a baby under their care. Some of these subtle practices often create an environment of shame and stigma, as seen below:

…where I work, we have a birth register that displays the outcomes of all childbirths month by month, and it is very easy to see the shifts where most babies are dying and the midwives on those shifts. Sometimes you see 38-, 39- and 40-weekers that died, and the first thing that comes to your mind is, 'what are the midwives doing?' No one wants their name on that list because it affects your credibility…other midwives avoid being on the same shift with you to protect their record. (P10)

## Support Needs of Midwives after a Stillbirth

This theme describes three subthemes: (1) lack of formal support, (2) need for moral and psychological support, and (3) need for improved working conditions.

### Lack of Formal Support

None of the participants received formal support from their employers following a stillbirth, and they were actually surprised by the question about support.

In Nigeria, there is nothing like that. The only support you can get is a pat on the shoulder from superiors that you tried your best. I don’t know of any other kind of support. (P3)

There is no formal support for stillbirths in my place of work. The only thing is that we encourage ourselves. We just tell ourselves that such things happen and are part of the experience. (P6)

The participants all said that having some support would be good.

### Need for Moral and Psychological Support

The participants acknowledged the personal and professional challenges associated with stillbirths, such as emotional imbalances, discouragement, blame and grief and called for moral support from colleagues and employers.

We need immediate support from superiors and co-workers to overcome the emotional imbalances associated with stillbirth. And people should not blame midwives. (P3)

I don’t think there is any support we can be given apart from moral and psychological support. We have signed up to be midwives. It is a part of our job. (P5)

As a midwife, you feel discouraged in such situations, so you need people around you to encourage you, especially senior colleagues and employers, to support you by giving you at least some time off the job to go home and grieve. We need encouragement to put the experience behind us. (P6)

### Need for Improvements in Working Conditions

When asked about how they can be best supported, most midwives focused on prevention and wanted the government and all relevant stakeholders to address the unfavourable working conditions that are contributing to many of the stillbirths.

We need our government to do more so that the high rates of stillbirth can be reduced. Sometimes we don't have essential resuscitation equipment, which could have averted some deaths…It is also part of support to train and re-train midwifery staff. Healthcare is dynamic, and things are changing every day. We need our practice to be current, and management needs to support us to be able to deliver for women and their families. (P3)

There should be some sort of support, especially in terms of staffing. We are really understaffed, and it is not optimal for one midwife and an attendant to man a busy ward. There's not much we can do since we are not magicians. If we have the adequate number of staff, we may be able to avoid some of these occurrences. The workload and stress are too much, it is not sustainable. They need to employ more hands. (P8)

## Discussion

The purpose of this study was to describe the experiences of Nigerian midwives who have experienced a stillbirth in their midwifery practice. The participants experienced sadness, guilt, shame, and emotional pain. These experiences were often aggravated by factors such as parental displays of grief, blame from parents and institutional inquiries into the loss. We also found that participants became more cautious in their practice, and felt humiliated by the experience. Despite the negative impact of stillbirth on the participants, they described their working environment as unsupportive. These findings are consistent with previous research (such as Bingham et al., 2023; Choummanivong et al., 2020; Finney et al., 2021).

One of the most compelling findings of this study is the negative perception of self and profession, even in situations where the cause of stillbirth was outside the control of the participants. The participants provided detailed explanations about how women’s delay in getting to the health facility and inadequate staffing contribute to stillbirth. Yet, they still questioned their competencies. This highlights how achieving a positive birth outcome has become an identifying characteristic of how midwives perceive themselves (McAra-Couper et al., 2014; McKellar et al., 2019). This is an important finding because it highlights how Nigerian midwives may be taking the blame for broader systemic failures, such as the underfunding of the healthcare system in Nigeria.

Similar to previous research (Bingham et al., 2023), the current study also found that participants were disappointed that their partnership with women did not yield a positive outcome. This finding reinforced that the relationship between midwives and women is central to the childbirth process and outcome. However, while the collaboration between midwives and parents is associated with positive pregnancy and childbirth experience, we found that it did not insulate midwives from parental blame and dissatisfaction with midwifery care. One explanation for this can be traced to the pluralistic nature of the maternity healthcare system in Nigeria, where midwives might not have formed a relationship with a woman until late in the pregnancy or during labour. This suggests that neither midwives nor parents in Nigeria will benefit from the partnership that ought to exist between midwives and parents. This calls for investment in sustainable models of midwifery that prioritise continuity of care.

Similar to earlier research (Finney et al., 2021; Mok et al., 2020), the participants identified better working conditions, moral support, and support from colleagues and employers as essential coping resources. Such desired forms of support can be used to plan supportive interventions for midwives in Nigeria. Making such support available may prevent issues such as dissatisfaction at work, burnout and intention to leave the profession, which have been seen among midwives who have experienced perinatal death in their practice (Garcia Catena et al., 2023).

## Strengths and Limitations

This is the first known study to explore midwives’ experiences of stillbirth in Nigeria, and it raises important issues regarding midwifery identity, professionalism, impact of stillbirth on midwives and the context in which stillbirths occur. However, despite variations in participants’ characteristics, the experiences uncovered in this study might not have captured the full range of midwives’ experiences of stillbirth. For example, Nigeria is made up of many tribes that are not represented in this study. Likewise, the participants in this study were also mid-career midwives. These factors limit the transferability of the findings to other settings. Future research needs to consider these limitations by employing methods such as surveys to investigate the issues raised in this study and be more intentional in recruiting larger and more diverse groups of midwives that can strengthen the case for generalisation of findings. The meaning of the ‘joy of midwives' needs further exploration, as it can provide information about job satisfaction, identity, burnout and turnover intention among midwives.

## Implications for Policy and Practice

This study reveals that the conditions in which Nigerian midwives work increase their risk for regular exposure to stillbirths, but they are not getting the support they need to deal with the impact of stillbirth. To make matters worse, the impact of stillbirth is not recognised in clinical practice, leaving midwives to rely on their own finite internal resources. Evidence is strong that a lack of support after encountering a perinatal loss can lead to burnout and high turnover intentions (Bingham et al., 2023; Finney et al., 2021). As such, policymakers, such as the Nursing and Midwifery Council of Nigeria, must recognise the impact of stillbirths on midwives and the risk it portends to the already fragile midwifery workforce in Nigeria. Health institutions employing midwives need to invest in moral and psychological support that is blame-free and that upholds professional pride. The Federal Ministry of Health must allocate more funding to address understaffing and equipment issues.

## Conclusion

The loss of a baby is a consequential event in midwifery practice because of its rippling effects on professional identity, which is the core of how midwives perceive themselves. It is important for health policy makers in Nigeria to listen to the needs of Nigerian midwives as captured in this study. This is important to enable them to cope with the multidimensional impact of stillbirth, while continuing to deliver high-quality midwifery care. Given the high rates of stillbirth in Nigeria, it is imperative that more is done to prevent stillbirths by strengthening midwifery workforce and improving working conditions of Nigerian midwives.
